# Supplementary material for: DEBKS: A Tool to Detect Differentially Expressed Circular RNAs
Source: Genomics Proteomics Bioinformatics. 2021 Feb 23;20(3):549–56. doi: 10.1016/j.gpb.2021.01.003 (PMC9801035; doi:10.1016/j.gpb.2021.01.003)
Supplement: Supplementary Table S1 — Primers used to validate differentially expressed circRNAs by RT-qPCR [file mmc1.docx]

**Table S1** **Primers used to validate differentially expressed circRNAs by RT-qPCR**

| **Target gene**  **(accession No.)** | **Position** | **ID** | **Primer** |
| --- | --- | --- | --- |
| *Nab1*  NM_008667 | circF | circ-Nab1-F | 5’-TGCCCTGCTGACAAGAAGAG-3’ |
|  | circR | circ-Nab1-R | 5’-CCAGGGTCCTAGGTAAGGCT-3’ |
|  | linearF | linear-Nab1-F | 5’-AGCGGAAGGAGAGTTTTGCT-3’ |
|  | linearR | linear-Nab1-R | 5’-GCTGGAAGAGTGTTGGCACA-3’ |
| *Pms1*  NM_153556 | circF | circ- Pms1-F | 5’-ACGGTGTCCTGAAACCTGAT-3’ |
|  | circR | circ- Pms1-R | 5’-CTCCGGCATCCAAGGAGTTT-3’ |
|  | linearF | linear- Pms1-F | 5’-GTGCAACTCAGGGTTTGCTG-3’ |
|  | linearR | linear- Pms1-R | 5’-CGACTGTTCACAGTGCTGCT-3’ |
| *Pde5a*  NM_153422 | circF | circ- Pde5a -F | 5’-GGGACATGTGGCAGCTTTTG-3’ |
|  | circR | circ- Pde5a -R | 5’-CTGCATGAGGACTTTGCTGC-3’ |
|  | linearF | linear- Pde5a -F | 5’-CTGGATGATCACCGGGACTT-3’ |
|  | linearR | linear- Pde5a -R | 5’-TCCCTGTGGTTCTTAATTGGCA-3’ |
| *N4bp1*  NM_030563 | circF | circ- N4bp1-F | 5’-AAAACGAACCAGGACGAGCA-3’ |
|  | circR | circ- N4bp1-R | 5’-TTGTGCCCCGACAAAAATGC-3’ |
|  | linearF | linear- N4bp1-F | 5’-GCGTATCTGGCTGCAACTCC-3’ |
|  | linearR | linear- N4bp1-R | 5’-ATGTTCCTGTTGCCGAGCTT-3’ |
| *Fam53b*  NM_001347630 | circF | circ- Fam53b -F | 5’-GTCCGGATGAAGTGCAGGAG-3’ |
|  | circR | circ- Fam53b -R | 5’-GCATTCCGGAGAGTGAGGTC-3’ |
|  | linearF | linear- Fam53b -F | 5’-GGACGACTCCAAGGCTACAC-3’ |
|  | linearR | linear- Fam53b -R | 5’-GATCGGGTACTGCTGACGAG-3’ |
| *Actb*  NM_007393 | linearF | Actb-F | 5'-TGTTACCAACTGGGACGACA-3' |
|  | linearR | Actb-R | 5'-GGGGTGTTGAAGGTCTCAAA-3' |
